# The CPCAT as a novel tool to overcome the shortcomings of NOEC/LOEC statistics in ecotoxicology: a simulation study to evaluate the statistical power

**DOI:** 10.1186/s12302-018-0178-5

**Published:** 2018-12-11

**Authors:** René Lehmann, Jean Bachmann, Bilgin Karaoglan, Jens Lacker, Glenn Lurman, Christian Polleichtner, Hans Toni Ratte, Monika Ratte

**Affiliations:** 1 FOM Hochschule für Oekonomie & Management, Herkulesstraße, Essen, Germany; 2 German Environment Agency, Wölitzer Platz, Dessau-Roßlau, Germany; 3 Private Scientist, Korsörer Straße, Berlin, Germany; 4 ToxRat Solutions GmbH & Co KG, Naheweg, Alsdorf, Germany

**Keywords:** LOEC, Generalized Poisson distribution, Species reproduction, Closure principle computational approach test (CPCAT)

## Abstract

Species reproduction is an important determinant of population dynamics. As such, this is an important parameter in environmental risk assessment. The closure principle computational approach test (CPCAT) was recently proposed as a method to derive a NOEC/LOEC for reproduction count data such as the number of juvenile Daphnia. The Poisson distribution used by CPCAT can be too restrictive as a model of the data-generating process. In practice, the generalized Poisson distribution could be more appropriate, as it allows for inequality of the population mean $\mu$ and the population variance $\sigma ^2$. It is of fundamental interest to explore the statistical power of CPCAT and the probability of determining a regulatory relevant effect correctly. Using a simulation, we varied between Poisson distribution ($\mu =\sigma ^2$) and generalized Poisson distribution allowing for over-dispersion ($\mu <\sigma ^2$) and under-dispersion ($\mu >\sigma ^2$). The results indicated that the probability of detecting the LOEC/NOEC correctly was $\ge 0.8$ provided the effect was at least 20% above or below the mean level of the control group and mean reproduction of the control was at least 50 individuals while over-dispersion was missing. Specifically, under-dispersion increased, whereas over-dispersion reduced the statistical power of the CPCAT. Using the well-known Hampel identifier, we propose a simple and straight forward method to assess whether the data-generating process of real data could be over- or under-dispersed.

## Introduction

In environmental risk assessment scientists often focus on assessing the effects of chemicals on an ecological system or specific environmental compartments [1]. Species reproduction considerably affects population dynamics and ecology. As such, the new closure principle computational approach test (CPCAT) was proposed for the evaluation of discrete reproduction data [2]. Numbers of new Lemna fronds (*Lemna minor L.*), numbers of juvenile Daphnids (*Daphnia magna*), and numbers of fish eggs laid are popular examples of reproduction count data. It is well known that count and proportion data in ecotoxicology are not normally distributed [3]. The reproduction data mentioned above are generally assumed to be Poisson distributed [4, 5].

Recently, a Poisson distribution together with CPCAT was used to test for differences in mean reproduction of different species [2]. Poisson distribution is known as the law of rare events [6]. Let $\mu$ represent mean reproduction and $\sigma ^2$ the variance. The Poisson model implies equality of mean reproduction and variance, see Eq. (1):

1 $$\begin{aligned} \mu =\sigma ^2. \end{aligned}$$

If a chemical substance affects mean reproduction, it affects variance, too. Consequently, effects on mean reproduction cause inhomogeneous variances among treatments. Furthermore, normal approximation of Poisson distributed data is only valid if mean reproduction $\mu \ge 5$ holds (e.g., mean numbers of laid eggs $\ge 5$). If a chemical substance reduces mean reproduction to near zero this normal approximation fails. Currently, the statistical power of CPCAT is unknown. As such, we conducted a simulation to assess how reliable the results obtained using CPCAT are. A key point of CPCAT is the assumption of a Poisson distribution. A Poisson distribution can be too conservative, as it implies expectation and variance to be equal [see Eq. (1)]. A generalized Poisson distribution allowing for over- and under-dispersion could be more appropriate. Using the Hampel identifier [7], we propose a simple and straight forward approach to assess whether observed data is over- or under-dispersed. The simulation included several scenarios of generalized Poisson distributed data and the statistical power of CPCAT was thus demonstrated. The probability of detecting the correct lowest observed effect concentration (LOEC) was also computed.

## Materials and methods

The LOEC is defined as the lowest treatment concentration at which an effect was seen, for example, reproduction differed statistically significantly from the control group. The NOEC is defined as the highest concentration at which no effect was seen. If the lowest tested concentration significantly affects reproduction, it is concordant with the LOEC and no NOEC can be derived as a consequence. We, therefore, used LOEC values instead of NOEC values.

### CPCAT: a short overview

The CPCAT is a combination of the closure principle (CP) [8] and the computational approach test (CAT) [9]. The mean reproduction of the control group is represented by $\mu _0$ and the mean reproduction of the *i*th treated group is represented by $\mu _i$.

**Fig. 1:**
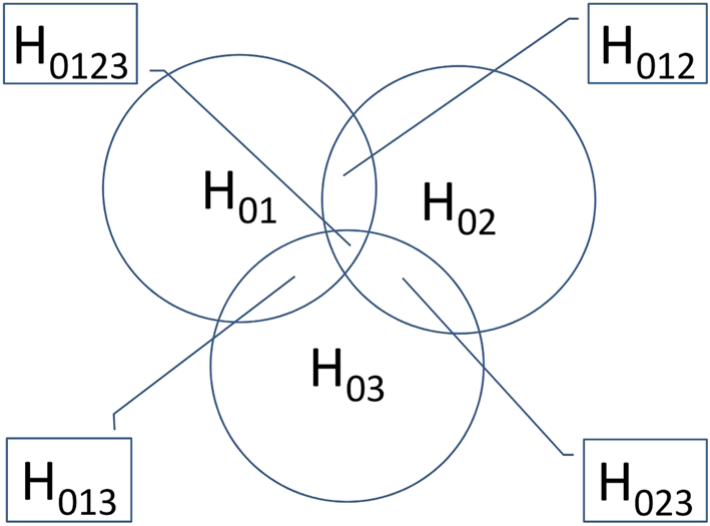
Illustration of the CP. Main null hypotheses $H_{0i}: \mu _0=\mu _i$ and intersection hypotheses

The CP is used to overcome $\alpha$-inflation, a major problem in multiple testing of “control vs. treatments”. It is illustrated in Fig. 1. The CP dictates that to test $H_{0}: \mu _0=\mu _1$, it is necessary to test $H_{0123}: \mu _0=\mu _1=\mu _2=\mu _3$, $H_{012}: \mu _0=\mu _1=\mu _2$, $H_{013}: \mu _0=\mu _1=\mu _3$, and $H_{01}: \mu _0=\mu _1$. As a consequence, the CPCAT is a two-sided computationally demanding test procedure, because more (intersecting) hypotheses have to be tested. On the other hand, $\alpha$-inflation is reduced to zero [8].

The CAT is used to test any (intersecting) hypothesis $H_{0,i_1,i_2,\ldots , i_r}: \mu _0=\mu _{i_1}=\mu _{i_2}=\cdots =\mu _{i_r}$, where $i_1, i_2,\ldots , i_r$ represent specific treatment groups. Instead of normal approximation, the CAT applies an estimated Poisson distribution of the reproduction count data. It is based on a parametric bootstrap, i.e., control and treatment data are re-sampled according to their estimated underlying Poisson distributions. Maximum Likelihood estimators (MLE) are used to compute the test statistic.

### Using the Hampel identifier as a pre-test for Poisson distribution

One feature of assuming a Poisson distribution is that the mean and variance are equal, see Eq. (1). If Eq. (1) is correct, then for the sample mean $\hat{\mu }$ and the sample variance $\hat{\sigma }^2$

2 $$\begin{aligned} \hat{\mu }\approx \hat{\sigma }^2 \end{aligned}$$

follows also. That is, ($\hat{\mu },\hat{\sigma }^2$)-pairs of Poisson distributed data should scatter around a line of slope = 1 with an intercept = 0 (identity line). To identify data sets not fulfilling Eq. (1) distances of ($\hat{\mu },\hat{\sigma }^2$)-pairs from the identity line (i.e., residuals) should be considered. Using the Hampel identifier and a cut-off value of 4.3 ($\alpha =0.01$) deviations from Eq. (1) can be determined [10]. Sachs [11] proposed a cut-off value of 5, independent of the sample size. Residuals exceeding the cut-off value correspond to ($\hat{\mu },\hat{\sigma }^2$)-pairs located far from the identity line (so-called outliers). Such ($\hat{\mu },\hat{\sigma }^2$)-pairs represent treatments that do not seem to fulfill Eq. (1). The underlying data-generating process is rather generalized Poisson. If $\hat{\sigma }^2-\hat{\mu }>5$ data are over-dispersed and if $\hat{\sigma }^2-\hat{\mu }<-5$ data are under-dispersed.

### The generalized Poisson distribution

Poisson distribution can be too conservative, because it requires expectation $\mu$ and variance $\sigma ^2$ to be equal. The generalized Poisson distribution allows for $\mu \ne \sigma ^2$. Let *X* be a generalized Poisson distributed random variable. The probability density function of *X* is presented in Eq. (3) [12, 13]:

3 $$\begin{aligned} P_{\theta ,\lambda }(X=x)={\left\{ \begin{array}{ll} \frac{\theta (\theta +x\lambda )^{x-1}}{x!}e^{-x\lambda -\theta } & x\in \mathbb N_0,\ \theta>0,\ \max \{-1,-\theta /m\}\le \lambda< 1\\ 0 &{} {\text{if}} \quad x>m, {\text{when}}\quad \lambda <0 . \end{array}\right. } \end{aligned}$$

The parameter $m\ge 4$ refers to the largest integer value satisfying $\theta +m\lambda >0$. Expectation and variance are given in Eqs. (4)–(5):

4 $$\begin{aligned} \mu=\, & {} \frac{\theta }{1-\lambda } \end{aligned}$$

5 $$\begin{aligned} \sigma ^2=\, & {} \frac{\theta }{(1-\lambda )^3}. \end{aligned}$$

Setting $\lambda =0$, we obtain the well-known Poisson distribution with $\mu =\sigma ^2=\theta$ [14]. If $\lambda <0$, the corresponding Poisson distribution is under-dispersed, that is $\sigma ^2<\mu$. Whereas, $\lambda >0$ implies over-dispersion represented by $\sigma ^2>\mu$ [12]. In the following, the term “Poisson distribution” refers to the case of $\mu =\sigma ^2$ if not otherwise stated. For further details concerning generalized Poisson distributions, refer to [15–17].

### Applicability of CPCAT in a generalized Poisson setting

Regarding the assumption of an underlying Poisson distribution the question arises whether CPCAT can be applied to generalized Poisson distributed data, too. A property of the Poisson distribution is that the population mean $\mu$ and the distribution parameter are equal. The MLE of $\mu$ under the null hypothesis is given by the sample mean. Moreover, the sample mean is an estimator of the first moment. Moment estimators are consistent and may be used as an approximation to MLE. They converge to the real underlying moments (e.g., population mean) as sample size increases and can be applied to every probability distribution [18]. Thus, CPCAT estimates the population mean $\mu$ approximately correct if the data-generating process is generalized Poisson distributed.

### The simulation

The R-package ZIGP (version 1.3) was used for the generation of (generalized) Poisson distributed data. ZIGP uses a re-parametrization of the generalized Poisson model allowing for larger over-dispersion factors than possible in the standard parametrization. For details, refer to [19].

All simulated trials consisted of one control group ($i=0$) and $k=4$ treatments, where each group contained 5 replicates. The number of simulations per scenario was $N=1000$.

We examined different situations of increasing and decreasing trends in $\mu _i$-values. Setting $\mu _0=\mu _1\ne \mu _2$ expectation of the control and treatment 1 are identical. As a consequence, an effect was given by treatment 2.

Values of $\mu _i$ varied among control and treatment groups. We chose $\mu _0\in \{25; 50; 75; 100; 125; 150\}$ and $\mu _2=a\cdot \mu _0$. For an increasing trend $a\in \{1.1; 1.2; 1.3; 1.4\}$, $\mu _3=1.5\mu _0$ and $\mu _4=1.7\mu _0$ was set. A multiplier of 1.7 was chosen, because multipliers larger than 1.7 tended to yield infinite simulated (generalized) Poisson distributed values preventing further statistical evaluations. For a decreasing trend $a\in \{0.9; 0.8; 0.7; 0.6\}$ was set, $\mu _3=0.5\mu _0$ and $\mu _4=0.1\mu _0$. Using this approach, the extent of the trend from the control group to treatment 3 was varied from slowly increasing/decreasing to rapidly increasing/decreasing.

Different $\sigma ^2=c\cdot \mu$ relations were investigated with $c\in \{0.1; 0.5; 1; 5; 10\}$, thereby accounting for under- and over-dispersion. Poisson distribution was obtained by setting $c=1$. Using $c<1$ or $c>1$, the data-generating process refers to an under- or over-dispersed generalized Poisson distribution, respectively. In total, 240 scenarios were simulated.

**Table 1:** PROB of the CPCAT (decreasing trend)

*c*	*a*	$\mu _0=25$	$\mu _0=50$	$\mu _0=75$	$\mu _0=100$	$\mu _0=125$	$\mu _0=150$
0.1	0.9	0	0.001	0	0.006	0.017	0.049
0.5	0.9	0.012	0.038	0.082	0.128	0.168	0.257
1	0.9	0.059	0.098	0.169	0.173	0.27	0.329
5	0.9	0.163	0.184	0.241	0.22	0.266	0.271
10	0.9	0.201	0.215	0.203	0.222	0.237	0.253
0.1	0.8	0.009	0.337	0.866	0.998	1	1
0.5	0.8	0.157	0.474	0.733	0.905	0.966	0.988
1	0.8	0.221	0.475	0.654	0.807	0.871	0.912
5	0.8	0.253	0.321	0.391	0.425	0.439	0.492
10	0.8	0.206	0.258	0.291	0.282	0.323	0.346
0.1	0.7	0.565	1	1	1	1	1
0.5	0.7	0.597	0.962	0.997	0.993	0.998	0.997
1	0.7	0.546	0.873	0.938	0.947	0.959	0.959
5	0.7	0.368	0.474	0.553	0.594	0.597	0.622
10	0.7	0.257	0.357	0.394	0.39	0.409	0.427
0.1	0.6	0.999	1	1	1	1	1
0.5	0.6	0.945	0.994	0.993	0.997	0.991	0.995
1	0.6	0.846	0.949	0.942	0.945	0.943	0.943
5	0.6	0.479	0.565	0.578	0.628	0.618	0.609
10	0.6	0.334	0.407	0.425	0.46	0.457	0.478

Parameter $c>(<)1$ indicates generalized Poisson distribution with over-(under-)-dispersion ($\sigma ^2=c\cdot \mu$) or Poisson distribution ($c=1$). Parameter *a* indicates the value of the true LOEC via $\mu _2=a\cdot \mu _0$. Parameter $\mu _0$ denotes the mean reproduction of the control group

**Table 2:** PROB of the CPCAT (increasing trend)

*c*	*a*	$\mu _0=25$	$\mu _0=50$	$\mu _0=75$	$\mu _0=100$	$\mu _0=125$	$\mu _0=150$
0.1	1.1	0	0	0	0.004	1	1
0.5	1.1	0.015	0.035	0.07	0.098	0.995	0.994
1	1.1	0.067	0.096	0.127	0.167	0.95	0.959
5	1.1	0.191	0.199	0.211	0.234	0.591	0.595
10	1.1	0.2	0.207	0.2	0.209	0.438	0.445
0.1	1.2	0.002	0.101	0.608	0.935	1	1
0.5	1.2	0.11	0.311	0.594	0.789	0.998	0.998
1	1.2	0.154	0.384	0.544	0.697	0.942	0.947
5	1.2	0.239	0.319	0.379	0.42	0.601	0.61
10	1.2	0.225	0.251	0.289	0.291	0.451	0.453
0.1	1.3	0.178	0.985	1	1	1	1
0.5	1.3	0.38	0.831	0.977	0.99	0.994	0.993
1	1.3	0.402	0.744	0.891	0.943	0.946	0.947
5	1.3	0.276	0.427	0.481	0.516	0.596	0.609
10	1.3	0.259	0.292	0.345	0.348	0.458	0.443
0.1	1.4	0.9	1	1	1	1	1
0.5	1.4	0.724	0.989	0.998	0.998	0.993	0.995
1	1.4	0.677	0.906	0.953	0.937	0.945	0.943
5	1.4	0.382	0.484	0.532	0.601	0.594	0.625
10	1.4	0.288	0.359	0.396	0.413	0.446	0.463

Parameter $c>(<)1$ indicates generalized Poisson distribution with over-(under-)-dispersion ($\sigma ^2=c\cdot \mu$) or Poisson distribution ($c=1$). Parameter *a* indicates the value of the true LOEC via $\mu _2=a\cdot \mu _0$. Parameter $\mu _0$ denotes the mean reproduction of the control group

### Results and discussion of the simulation

The simulation was designed such that $\mu _0=\mu _1\ne \mu _2$. That is, the lowest effect concentration (LEC) refers to treatment 2. Thus, the LOEC derived using CPCAT should equal the LEC. PROB is defined to be the probability of the event “LOEC=LEC”, that is, the probability of deriving the correct LOEC. PROB values are presented in Tables 1, 2. The significance level was set to $\alpha =0.05$.

It can be seen that PROB was larger for rapidly increasing/decreasing trends between the control group and treatment 3 than for slowly. This result is not surprising, because the larger the difference between $\mu _0$ and $\mu _2$ the more easily the true LOEC can be derived.

**Fig. 2:**
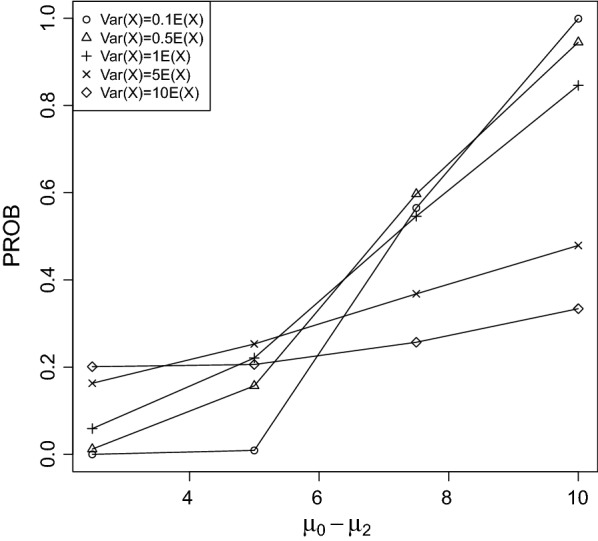
PROB of the CPCAT; $\mu _0=25$, decreasing trend

**Fig. 3:**
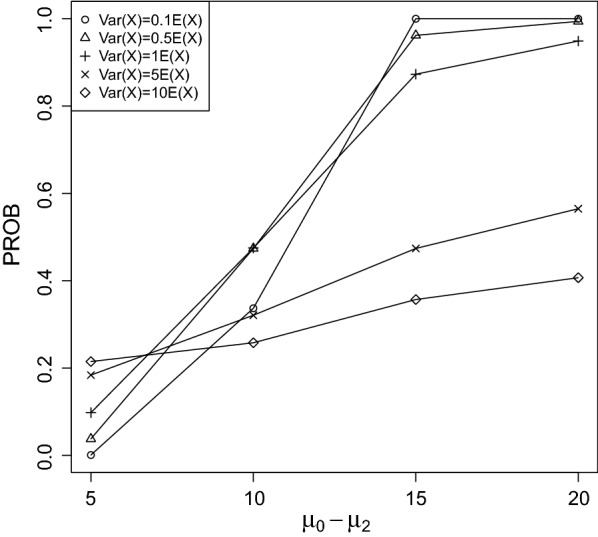
PROB of the CPCAT; $\mu _0=50$, decreasing trend

**Fig. 4:**
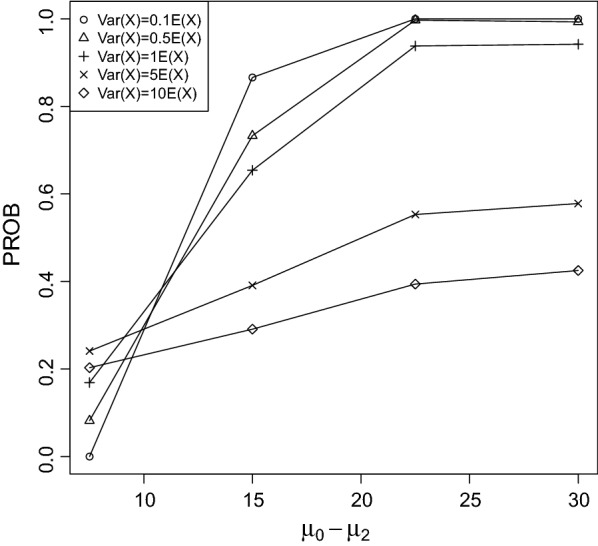
PROB of the CPCAT; $\mu _0=75$, decreasing trend

**Fig. 5:**
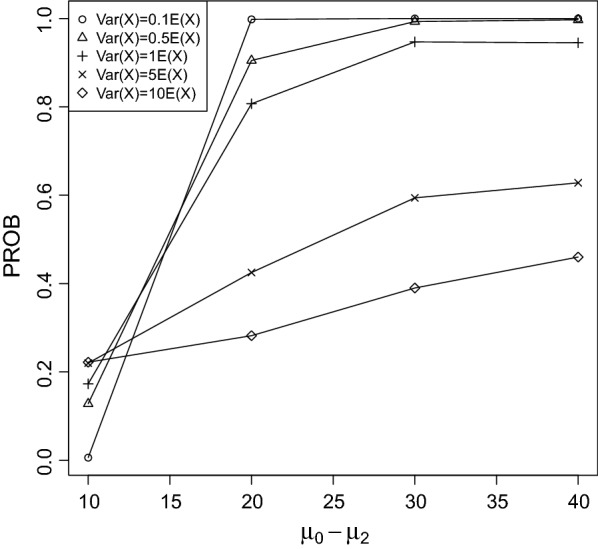
PROB of the CPCAT; $\mu _0=100$; decreasing trend

**Fig. 6:**
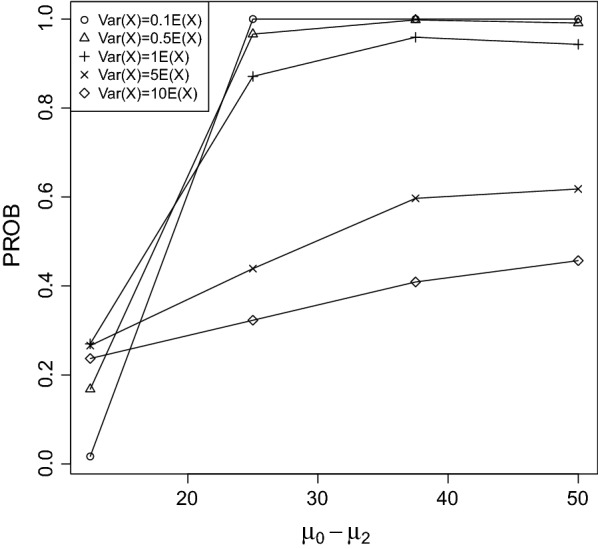
PROB of the CPCAT; $\mu _0=125$, decreasing trend

**Fig. 7:**
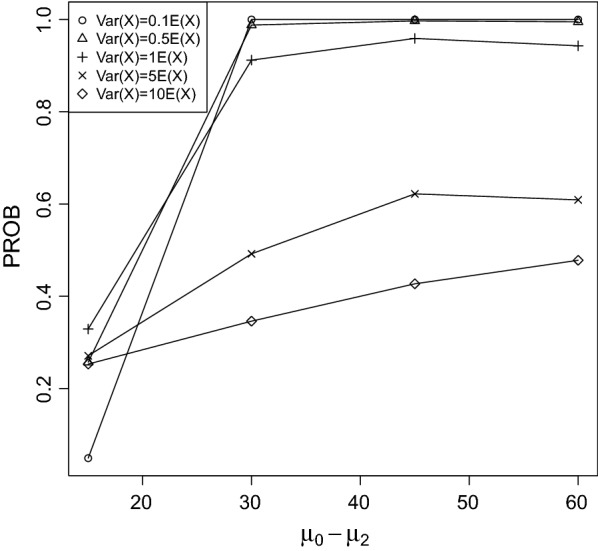
PROB of the CPCAT; $\mu _0=150$, decreasing trend

**Fig. 8:**
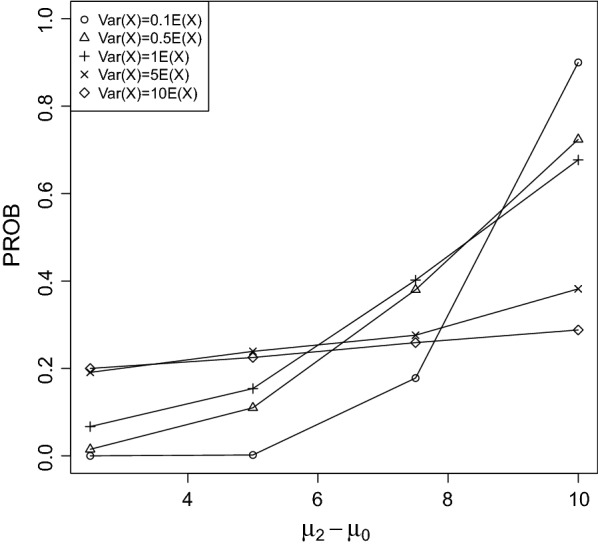
PROB of the CPCAT; $\mu _0=25$, increasing trend

**Fig. 9:**
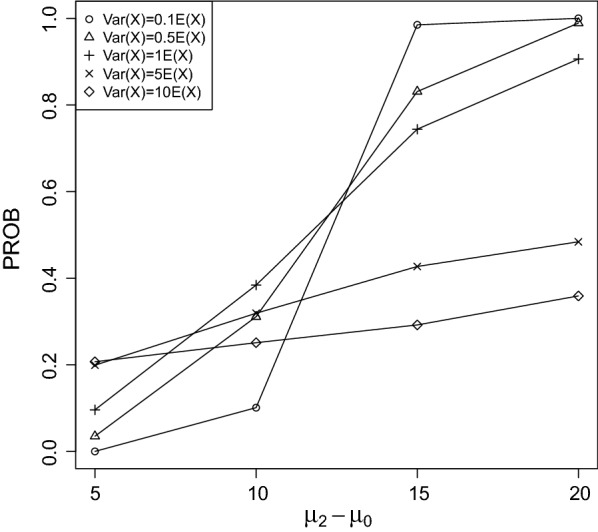
PROB of the CPCAT; $\mu _0=50$, increasing trend

**Fig. 10:**
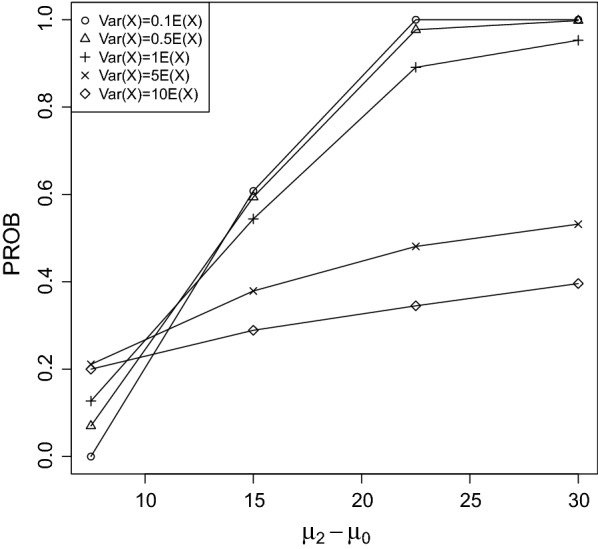
PROB of the CPCAT; $\mu _0=75$, increasing trend

**Fig. 11:**
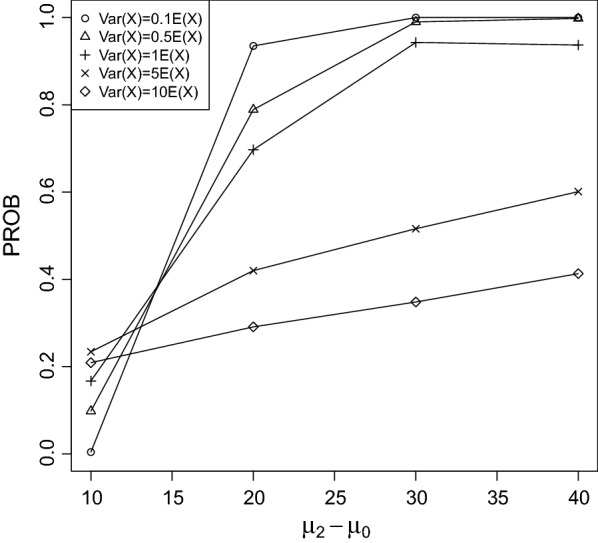
PROB of the CPCAT; $\mu _0=100$, increasing trend

**Fig. 12:**
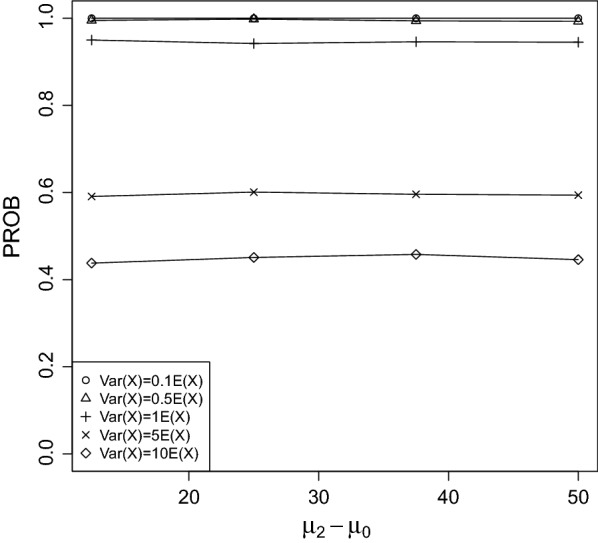
PROB of the CPCAT; $\mu _0=125$, increasing trend

**Fig. 13:**
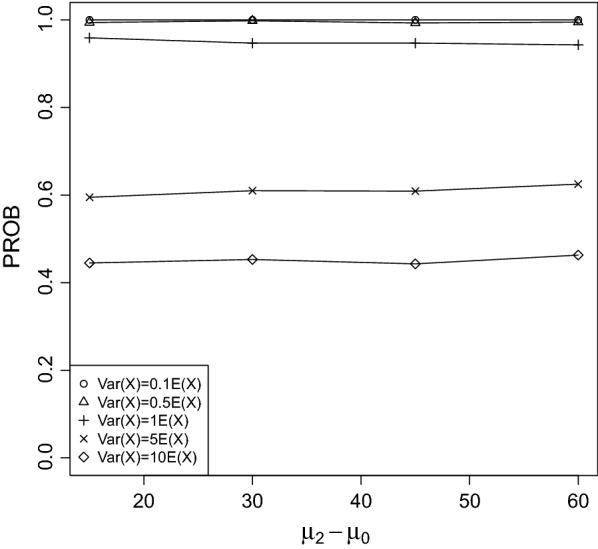
PROB of the CPCAT; $\mu _0=150$, increasing trend

PROB is illustrated in Figs. 2, 3, 4, 5, 6, 7, 8, 9, 10, 11, 12 and 13. The individual differences given on the x-axis for the data points generally correspond to 10%, 20%, 30%, and 40% effect. Over-dispersion (under-dispersion) increases (decreases) the probability of observing overlapping treatment data reducing (increasing) PROB. Simulation results indicated that ${\text {PROB}}_{\sigma ^2<\mu }>{\text {PROB}}_{\sigma ^2=\mu }>{\text {PROB}}_{\sigma ^2>\mu }$ was correct, i.e., the statistical power of the CPCAT increases if the underlying generalized Poisson distribution is under-dispersed. On the other hand, over-dispersion reduces statistical power.

PROB depends on various parameters, namely, direction of the observed trend, magnitude of the effect, and steepness of the observed effects. PROB is larger for decreasing than for increasing trends. This is due the characteristics of the Poisson distribution. For $\mu _0\le 75$, a difference $|\mu _0-\mu _2|\ge 15$ provides PROB of approximately 0.8. For $\mu _0\ge 100$, a difference $|\mu _0-\mu _2|\ge 18$ is required to obtain PROB $\approx 0.8$. Thus, PROB $\ge 0.8$ if $|\mu _0-\mu _2|\ge 0.2\mu _0$ and $\mu _0>50$ while over-dispersion is missing.

Mean reproduction varies between species. It can be low (e.g., number of laid bird eggs) or large (e.g., reproduction of Collembola *Folsomia candida*). We tried to cover a large range of mean reproduction values using a set of many different $\mu$ values. However, a simulation using a mean reproduction level greater than $150\times 1.7=255$ could not be applied, because the number space of a computer is limited and infinite values were generated for $\mu >255$ (see “The simulation” section).

For some species, it could be appropriate to assume over- or under-dispersion of reproduction data. We tried to choose a realistic range of dispersion factors $c\in \{0.1; 0.5; 1; 5; 10\}$. For example, in some real data, we found reproduction of Collembola (*Folsomia candida*) being over-dispersed by factor 10. On the other hand, if a substance reduces reproduction to nearly 0 dispersion will be reduced to nearly 0, too. Thus, a factor of 0.1 can be reasonable, too.

## Conclusion

Statistical theory and results of the simulation indicated that the CPCAT is applicable and powerful provided $\mu =\sigma ^2$ or $\mu >\sigma ^2$ holds. In the case of $\mu <\sigma ^2$, the statistical power is reduced.

To determine whether or not the data are over- or under-dispersed, the cut-off value of the Hampel identifier, as explained in “Using the Hampel identifier as a pre-test for Poisson distribution” section should be used. The difference between any pair $(\hat{\mu }, \hat{\sigma }^2)$ and the identity line is given by $\hat{\sigma }^2-\hat{\mu }$. The Hampel identifier is relevant in real data analyses, because it can indicate over-dispersion. Over-dispersion reduces the probability of detecting regulatory relevant effects. From the simulation, we can estimate the loss of statistical power.

Overall, the CPCAT is applicable to generalized Poisson distributed data. A future version of CPCAT must explicitly take into account over- and under-dispersion, e.g., using MLE of the distribution parameters $\theta$ and $\lambda$.

## Abbreviations

CAT: computational approach test
CP: closure principle
CPCAT: closure principle computational approach test
LOEC: lowest observed effect concentration
MLE: maximum likelihood estimator
LEC: lowest effect concentration
NOEC: no observed effect concentration
PROB: probability of correct derivation of the LOEC
